# Considering Factors in the Single- Versus Dual-coil Lead Debate

**DOI:** 10.19102/icrm.2018.091008

**Published:** 2018-10-15

**Authors:** Claudio Tondo

**Affiliations:** ^1^Heart Rhythm Center at Monzino Cardiac Centre, IRCCS Department of Clinical Sciences and Community Health University of Milan, Milan, Italy

**Keywords:** Implantable cardioverter-defibrillator, inappropriate shock, lead extraction

The case report by Gul et al.^1^ is well-written and reports a potential untoward effect promoted by the implantation of a dual-coil implantable cardioverter-defibrillator (ICD) lead.

A close observation of the patient’s chest X-ray **(Figure 1B)** provided by the authors reveals that the superior vena cava (SVC) coil is shifted downward, close to the tricuspid valve. Therefore, it appears that the SVC coil was already approaching the tricuspid valve by the postprocedure time.

The information provided by this clinical case is undoubtedly crucial, highlighting the relevance of encouraging the implantation of single-coil ICDs so as to reduce the potential of such a complication. Though recent research suggests that the implantation of dual-coil ICDs could still be more popular at this time in clinical practice,^2^ according to one meta-analysis, dual-coil ICDs may demonstrate higher rates of lead-related complications and all-cause mortality.^3^ Separately, when used for primary prevention in patients without indications for pacing, dual-chamber devices were associated with a higher risk of device-related complications and similar one-year mortality and hospitalization outcomes versus single-chamber devices.^4^

In the case of a need for lead extraction, single-coil ICD leads may be easier to remove due to the greater tension required to successfully extract dual-coil ICD leads.^5^ Dual-coil ICD implantation with SVC coil placement may also further increase the difficulty of (by 2.6-fold) and risk of complications during lead extraction.^6^

Varying data exist regarding the inappropriate shock rates of single-coil and dual-coil devices, though a number of studies suggest there is no significant difference between the two.^7–10^ However, the other aforementioned factors support the thought posed by Gul et al.,^1^ that perhaps single-coil ICDs should be considered first for implantation before dual-coil devices wherever appropriate.
